# Longitudinal Analysis of Macronutrient Composition in Preterm and Term Human Milk: A Prospective Cohort Study

**DOI:** 10.3390/nu11071525

**Published:** 2019-07-04

**Authors:** Céline J. Fischer Fumeaux, Clara L. Garcia-Rodenas, Carlos A. De Castro, Marie-Claude Courtet-Compondu, Sagar K. Thakkar, Lydie Beauport, Jean-François Tolsa, Michael Affolter

**Affiliations:** 1 Clinic of Neonatology, Department Woman Mother Child, University Hospital of Lausanne, 1011 Lausanne, Switzerland; 2 Nestlé Institute of Health Sciences, Nestlé Research, 1000 Lausanne, Switzerland; 3 Clinical Development Unit, Nestlé Research Asia, Singapore 138567, Singapore; 4 Nestlé Institute of Food Safety & Analytical Science, Nestlé Research, 1000 Lausanne, Switzerland; 5 Nestlé Research Asia, Singapore 138567, Singapore

**Keywords:** human milk, preterm, term, neonate, infant, macronutrients, protein, fat, lactose, nutrition

## Abstract

Background: Mother’s own milk is the optimal source of nutrients and provides numerous health advantages for mothers and infants. As they have supplementary nutritional needs, very preterm infants may require fortification of human milk (HM). Addressing HM composition and variations is essential to optimize HM fortification strategies for these vulnerable infants. Aims: To analyze and compare macronutrient composition in HM of mothers lactating very preterm (PT) (28 0/7 to 32 6/7 weeks of gestational age, GA) and term (T) infants (37 0/7 to 41 6/7 weeks of GA) over time, both at similar postnatal and postmenstrual ages, and to investigate other potential factors of variations. Methods: Milk samples from 27 mothers of the PT infants and 34 mothers of the T infants were collected longitudinally at 12 points in time during four months for the PT HM and eight points in time during two months for the T HM. Macronutrient composition (proteins, fat, and lactose) and energy were measured using a mid-infrared milk analyzer, corrected by bicinchoninic acid (BCA) assay for total protein content. Results: Analysis of 500 HM samples revealed large inter- and intra-subject variations in both groups. Proteins decreased from birth to four months in the PT and the T HM without significant differences at any postnatal time point, while it was lower around term equivalent age in PT HM. Lactose content remained stable and comparable over time. The PT HM contained significantly more fat and tended to be more caloric in the first two weeks of lactation, while the T HM revealed higher fat and higher energy content later during lactation (three to eight weeks). In both groups, male gender was associated with more fat and energy content. The gender association was stronger in the PT group, and it remained significant after adjustments. Conclusion: Longitudinal measurements of macronutrients compositions of the PT and the T HM showed only small differences at similar postnatal stages in our population. However, numerous differences exist at similar postmenstrual ages. Male gender seems to be associated with a higher content in fat, especially in the PT HM. This study provides original information on macronutrient composition and variations of HM, which is important to consider for the optimization of nutrition and growth of PT infants.

## 1. Introduction

Human milk (HM) is a highly complex, dynamic and species-specific system that incorporates numerous nutritional and bioactive elements. Despite progress in analytical technologies enabling the growth of data over the past decade, HM composition remains only partially elucidated [1,2,3,4]. Recent studies revealed countless inter- and intra-individual factors influencing HM composition, such as maternal, circadian, pregnancy, delivery, infantile, chronological and environmental variables [5,6,7,8,9]. Among these factors, temporal changes of HM composition before and after term equivalent time are of particular interest considering the critical challenges of preterm (PT) infants’ nutrition and the critical importance of HM for this vulnerable population [10,11].

As for healthy term (T) babies, breastfeeding provides numerous health advantages for PT infants and their mothers, and is thus strongly recommended [12,13,14,15,16]. In addition, for very PT at risk neonates, mother’s own milk appears particularly protective against several severe complications, such as necrotizing enterocolitis, sepsis, bronchopulmonary dysplasia, retinopathy of prematurity, and reduces duration of hospital stay or incidence of rehospitalizations [17,18,19,20]. Furthermore, it may improve brain growth and the development of these fragile infants [21,22,23,24].

Despite these crucial protective effects, HM may not fully meet some specific additional nutritional requirements of very PT infants to avoid growth flattening, nutritional deficits, and related complications [25,26,27]. Therefore, human milk fortification is often recommended in very PT neonates, however controversies exist and optimal, feasible fortification strategies remain to be identified [11,16]. Nevertheless, even with the development of HM fortification practices, growth failure remains frequent in neonatal intensive care units (NICUs), affecting up to half of very low birthweight neonates [28].

In this context, we need to have better knowledge and understanding of HM composition and its variations, and especially temporal changes, in order to enhance nutritional enteral strategies, mainly human milk fortification and/or formula complements according to the situation [11]. Currently, studies on HM composition after very PT delivery remain scarce and are mostly based on cross-sectional studies. Only a few studies have longitudinally measured the trajectory of HM composition and its time variations in both T and PT infants, and the data remain controversial [10].

In this prospective cohort study, we aimed: (i) to quantify macronutrient composition in very PT and T HM over time; (ii) to compare macronutrient composition between PT and T milk, at similar lactation stages (postnatal ages) and gestational stages (postmenstrual ages); and (iii) to investigate other factors potentially associated with macronutrient variations in HM.

## 2. Materials and Methods 

### 2.1. Study Design, Subjects, and Setting

This study was a part of a prospective, monocentric, cohort study aiming to analyze various components of HM over time and to compare their contents in the PT HM versus the T HM. We previously published the study design and the description of the population in detail [29]. 

The study was conducted between October 2013 and July 2014 at the University Hospital of Lausanne, Switzerland. Eligible mothers were those older than 18 years of age, intending to breastfeed their offspring, and who delivered either (i) very prematurely (PT group), from 28 0/7 to 32 6/7 weeks of gestational age (GA) or (ii) at term (T group), between 37 0/7 to 41 6/7 weeks of GA.

Exclusion criteria included any counter-indication to breastfeeding, maternal diabetes (type I or II) before pregnancy, alcohol or drug consumption, and insufficient French language skills.

The mothers who were included in the study were followed until postnatal week 16 for the PT group, and week 8 for the T group, or until lactation discontinuation (whichever came first). 

### 2.2. Data Collection

The main clinical and sociodemographic maternal and neonatal characteristics were prospectively collected. All data were recorded in electronic case report forms in a dedicated database.

### 2.3. Milk Sampling and Processing

**Figure 1:**
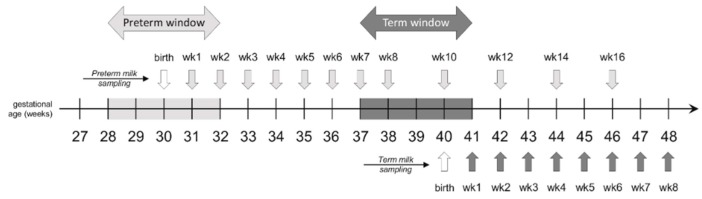
Milk sampling schedule (reprinted from Garcia-Rodenas et al. 2018, with permission from Elsevier).

Sequential, iterative samples of milk were collected according to the following schedule, illustrated in Figure 1:

(i) Preterm group: 1–10 mL of milk was collected at the end of the 1st week, then weekly for the first 8 weeks, then every 2 weeks for an additional 8 weeks; this corresponded thus to a maximum of 12 samples during the 16 weeks. 

(ii) Term group: 10 mL of milk were collected weekly for 8 weeks, starting at the end of week 1. 

Standardized milk sampling relied on a single sampling of a single breast in the morning (first morning expression). Milk samples were expressed between 6–12 a.m., using an electric breast pump (Symphony^®^, Medela, Baar, Switzerland). After the breast was entirely emptied, the milk was homogenized and an aliquot of maximum 10 mL of milk (1–10 mL for the first two time points in the PT group) was collected. Each sample aliquot was immediately transferred to dedicated freezing tubes (15 mL polypropylene tubes, Falcon™, Fisher Scientific, Reinach, Switzerland), and stored at −18 °C in the home freezer for a maximum of 1 week until frozen transfer to the hospital. At the hospital, samples were temporarily kept at −80 °C and then transferred to the Nestlé Research Centre (NRC, Lausanne, Switzerland). The frozen milk samples were thawed for splitting into 15 aliquots and stored at −80 °C until analysis of macronutrient and other HM components (reported in independent publications).

### 2.4. Measurement of HM Macronutrient Composition

The macronutrient content (total protein, fat, and carbohydrate, i.e., lactose) and energy density were measured using a human milk analyzer (MIRIS AB, Uppsala, Sweden) based on mid-infrared transmission spectroscopy (www.mirissolutions.com). One mL of each milk sample was thawed and warmed to 40 °C in a water bath. Prior to the measurement, each sample was homogenized using the MIRIS sonicator to avoid protein aggregation and lipid phase separation. A daily calibration check (MIRIS check solution) was performed according to the manufacturer’s recommendations. Macronutrient quantities were measured in a single run and the energy content was calculated automatically by the MIRIS instrument (protein 4.4 kcal/g, carbohydrate 4 kcal/g, fat 9 kcal/g) [30]. 

We used a MIRIS measure validation process that had been previously published [31]. As in other literature reports, this validation showed that the MIRIS was not accurate enough for the measurement of total protein in human milk as compared with the Kjeldahl reference method (AOAC International, method number 991.22), while no differences were observed for fat and carbohydrate content as compared with the corresponding reference methods. To address this issue, we used colorimetric bicinchoninic acid (BCA, ThermoFisher Scientific) assay, which produced accurate human milk protein values as compared with those obtained with the reference Kjeldahl method. In order to include this more accurate protein information in the energy density value of the human milk samples, energy density was recalculated for each milk sample using the same formula as the MIRIS instrument: Energy (kcal/L) = protein (g/L) × 4.4 + fat (g/L) × 9.25 + carbohydrate (g/L) × 4. 

### 2.5. Statistics

The paucity of quantitative data on the macronutrient content in the PT HM precluded a proper power calculation in this exploratory study. The study size was initially set at *n* = 20 subjects per group (preterm and term infant mothers) according to the estimated recruitment feasibility at the study center within a one-year period.

The temporal changes of macronutrient contents were compared in the PT and T HM at equivalent infant (1) postpartum ages and (2) postmenstrual ages. For both comparisons, mixed linear models were used to estimate the differences between preterm and term infants. The models used age (either postpartum or postmenstrual), term/preterm status, and interaction between age and term/preterm status. Within subject variability was accounted for by declaring the subject ID as a random effect. Contrast estimates of the model were calculated by comparing the PT and T HM groups at each time point. No imputation method was applied for missing data (both in between visits and loss to follow up) as the method used does not require a complete dataset. A conventional two-sided 5% error rate was used without adjusting for multiplicity as this exploratory trial is for hypothesis generation purposes.

Similar methods were used to analyze the effects of gender and delivery mode and their interaction with age (both postpartum and postmenstrual and also both in the PT and the T populations together and separately). Statistical analyses were done using SAS 9.3 (SAS Institute, Cary, North Carolina, USA) and R 3.2.1 (R Foundation for Statistical Computing, Vienna, Austria; https://www.R-project.org).

### 2.6. Ethics

The study was approved by the local Ethical Board (Commission cantonal d’éthique de la recherche sur l’être humain du Canton de Vaud) (Protocol 69/13, clinical study 11.39.NRC; April 9, 2013). Maternal written consent was obtained. The study was registered at ClinicalTrials.gov with the identifier NCT02052245.

## 3. Results

### 3.1. Study Population 

The detailed study flow chart and study population description has been published elsewhere [29]. They are presented in the supplementary material (see Figure S1 and Table S1).

Sixty-one mothers were included in the study: 27 mothers of 33 PT neonates, and 34 mothers of 34 T neonates. In the PT group, 25/27 mothers (93%) completed the study (one neonatal death, one withdrawal). In the T group, 28/34 mothers (82.4%) completed the study (one maternal illness, two withdrawals, three early breastfeeding disruption). In all, we collected 500 HM samples (280 PT and 220 T samples). 

Mothers of the two groups were comparable in age (mean ± SD: 32.4 ± 5.6 years in the PT group, versus 31.2 ± 4.2 years in the T group, *p* = 0.3173) and body mass index before pregnancy and at delivery. The mean ± SD gestational age and birthweight of the PT and T neonates differed as expected (30.8 ± 1.4 weeks versus 39.5 ± 1.0 weeks, *p* < 0.0001, and 1421 ± 373 g versus 3278 ± 354 g, *p* < 0.0001), and more PT neonates were born by caesarean section (63% versus 23%, *p* = 0.0019). The sex ratio was similar in the two groups (54% versus 53% of males, *p* = 0.8952).

### 3.2. HM Macronutrient Composition

Overall, we noticed a substantial intra- and inter-individual variability in data for all macronutrients, indicating a large heterogeneity between macronutrient content for milk samples. Maximal/minimal ratios reached up to 16 for fat, 5.4 for protein, 4.1 for energy, and 3.4 for lactose content. Detailed numerical values of each macronutrient according to postnatal age (A) and postmenstrual age (B) are reported in the corresponding supplemental Tables.

#### 3.2.1. Total Protein

**Figure 2:**
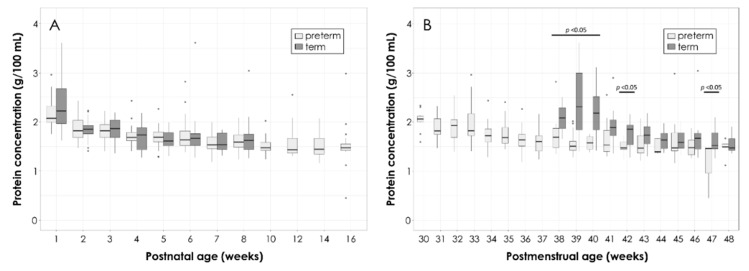
Total protein content in the preterm (PT) and the term (T) human milk (HM) according to postnatal age (**A**) and postmenstrual age (**B**). Box plots represent medians with 25th and 75th percentile, minimal-maximal range, and outliers (values >4.5 are excluded from the graph). Statistically significant *p*-values are indicated in the graph.

The values of total protein content in the PT and T HM according to postnatal age are depicted in Figure 2A. The total protein content gradually decreases from birth to four months, from (mean ± SD) 2.2 ± 0.3 g/100 mL to 1.5 ± 0.5 g/100 mL in preterm and from 2.5 ± 0.8 g/100 mL to 1.7 ± 0.3 g/100 mL in T HM, without significant differences between the PT and T groups at any point in time. 

Figure 2B shows the total protein content in the PT and T HM according to postmenstrual age. There were significant differences (*p* < 0.005) at gestational ages of 38–40 and 42 weeks, with T HM containing higher amounts of protein. These differences peaked at 39 weeks with T values up to 1.7 times higher than PT values. Later postmenstrual ages (>43 weeks) did not show any significant differences in protein content.

#### 3.2.2. Total Fat

**Figure 3:**
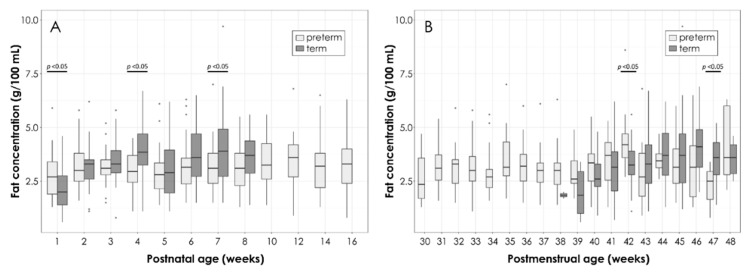
Total fat content in PT and T HM according to postnatal age (**A**) and postmenstrual age (**B**). Box plots represent medians with 25th and 75th percentile, minimal-maximal range, and outliers. Statistically significant *p*-values are indicated in the graph.

The mean values for total fat content in the PT and T HM slightly increase in early lactation and then remain constant over time (Figure 3A). As compared to the T HM, the PT HM contained significantly more fat (mean ± SD, 2.8 ± 1.1 g/100 mL in PT HM versus 2.1 ± 1.0 g/100 mL in T HM; *p* = 0.04) in the first week of lactation, but less in the later stages of lactation (week three to eight). 

When comparing the fat content according to postmenstrual age (Figure 3B), two significant differences were observed for gestational age of 42 and 47 weeks, respectively, with higher fat content at week 42 and lower fat content at week 47 in the PT HM as compared to the T HM.

#### 3.2.3. Total Lactose

**Figure 4:**
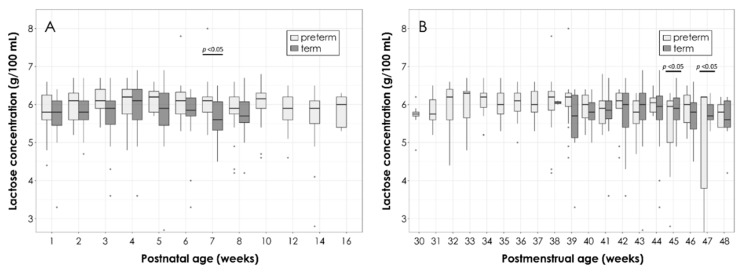
Total lactose content in the PT and T HM according to postpartum age (**A**) and postmenstrual age (**B**). Box plots represent medians with 25th and 75th percentile, minimal-maximal range, and outliers. Statistically significant *p*-values are indicated in the graph.

The mean values for lactose content remained stable over postnatal time in both groups with a consistent, but not significant, higher content in the PT versus the T HM (mean ± SD over all lactation stages: 5.9 ± 0.2 g/100 mL versus 5.8 ± 0.1 g/100 mL) (Figure 4A). A significant difference was observed at week 7 with the PT HM containing higher amounts of lactose (6.1 ± 0.5 g/100 mL versus 5.6 ± 0.9 g/100 mL, *p* = 0.0233). 

When comparing lactose contents according to gestational age (Figure 4B), significant variations were found for week 45 and 47, where lactose content was lower in the PT HM than in the T HM.

#### 3.2.4. Energy Density

**Figure 5:**
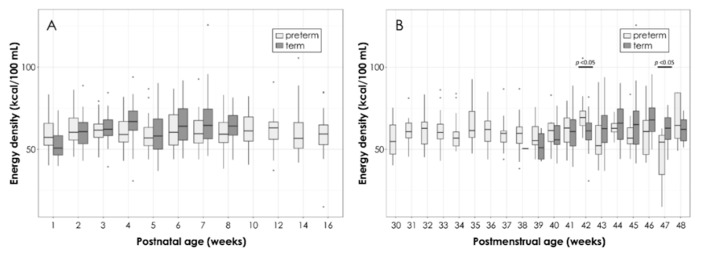
Calculated energy density in the PT and T HM according to postpartum age (**A**) and postmenstrual age (**B**). Box plots represent medians with 25th and 75th percentile, minimal-maximal range, and outliers. Statistically significant *p*-values are indicated in the graph.

The mean ± SD values for energy density tended to be higher in the PT HM (58.7 ± 10.2 kcal/100 mL versus 53.1 ± 8.8 kcal/100 mL; *p* = 0.08) in the first week of lactation, whereas in weeks three to eight postpartum, energy density in the T HM was higher (Figure 5A). 

When comparing the energy density according to postmenstrual age (Figure 5B), there were punctual variations between the PT and T HM, but trajectory over lactation remained relatively constant. 

#### 3.2.5. Other Factors Influencing HM Macronutrient Composition

The composition of macronutrients was also compared over time in the population and in each T and PT subgroup separately, according to infant gender or mode of delivery (see Figures in supplementary material).

Milk of mothers with male infants was consistently higher in fat and energy as compared to milk of mothers with female infants, reaching statistical significance at postnatal weeks five and seven (estimated differences of 0.89 g/100 mL and 1.07 g/100 mL of fat, respectively). The difference at five weeks was more pronounced in the PT group (estimated difference of 1.04 g/100 mL) and the difference at seven weeks was more pronounced for the T group (1.58 g/100 mL). There was also a trend of increased content of total protein in milk for male as compared to female infants in both the T and the PT groups, but the differences were not significant. We did not observe a gender difference for lactose content. Regarding the mode of delivery, it was not associated with consistent nor significant changes in HM macronutrients contents in our population.

A mixed linear model, taking into account the variables T or PT, postpartum age, interaction between T/PT and postpartum age, gender, and mode of delivery, was assessed in order to have an indication of which variables affect macronutrient concentration. It was observed that in general, the postpartum age has a significant effect on the macronutrient concentration of milk. Carbohydrate content was significantly higher for premature infants globally (all time points combined) while differences in proteins, energy, and fat depended on the postpartum age. Energy and fat concentration were significantly affected by the gender of the infant.

## 4. Discussion

This study provides substantial longitudinal data on the macronutrient compositions of 500 HM samples from 61 mothers delivering either very prematurely (*n* = 27) or at term (*n* = 34), during a time period of up to 16 and eight weeks for a PT and a T group, respectively. The original milk sampling design enabled the comparison of the PT and T HM composition not only at similar postnatal ages, corresponding to similar maternal lactation stages, but also at similar postmenstrual ages, corresponding to a similar infant developmental stage. 

In this cohort, HM iterative macronutrients analysis showed notably: (i) large inter- and intra-subject variations, most marked for fat content; (ii) little differences between the PT and T HM at similar postnatal ages/lactation stages, while there were more significant differences between the PT and T HM at similar postmenstrual ages/developmental stages; (iii) an overall decrease in total protein content over time, whereas lactose, fat, and energy density remained stable; and (iv) some gender differences in HM composition, as HM dedicated to male infants tended to be richer in fat and energy.

First, we have emphasis on the magnitude of the variations observed in our cohort. Differences between minimal and maximal measurements reached up to more than 10 times for fat. These variations are more important, on the whole, than those reported in other studies [10,11,32]. Extreme values were verified and confirmed. An effect of milk preservation or homogenization is unlikely, as the procedure was standardized [33]. An impact of the method of collection (first morning one single breast, rather than 24 h two breasts collections) is possible [5]. Nevertheless, these results confirm and underline how much a unique “standard” HM composition basis of calculation cannot fit all individual situations, and that more accurate alternatives for estimations of nutritional intake are required, notably for fortification issues [34].

Among factors of variations, temporal changes in HM macronutrient composition have been previously reported [10,35,36,37]. The study plan allowed us to investigate the HM composition changes according to both postnatal and gestational ages, related to adaptive and developmental stages, respectively. Interestingly, in our study, macronutrient composition changes were overall more pronounced according to postnatal time than to gestational age, which is in line with other recent observations [38]. Consequently, there were fewer differences between the PT and the T HM at similar postnatal ages, than at similar gestational ages. Its final implication, however, remains to be further explored. This finding should also be considered regarding the definition of nutritional needs of the PT infants and establishment of fortification targets.

Concerning the protein composition, the observed trajectory in our cohort follows a progressive decrease, which is consistent with previous observations [10,35,39]. Our protein values align well with published results for the first four weeks of lactation, and then seem to remain slightly higher than reported values (week 6 to 12) [10,37]. Unlike most existing data, we did not find a higher protein content in the PT HM than in the T HM, and there were no significant differences in the PT and T HM protein contents at any point. By contrast, the longitudinal trajectory of the protein content according to postmenstrual age revealed some significant differences between PT and T HM, which culminate between weeks 38 and 42. This implies that a preterm breastfed infant arriving at term equivalent age receives lower amounts of protein as compared with a breastfed term infant.

Concerning the fat composition, the changes of the PT and T HM contents follow a very similar trajectory as described previously, with an initial slight increase in fat content from week one to week two, followed by a constant level over the remaining lactation period. Initially at week one, the PT HM contained a higher fat content, and then, until the end of the sampling period, the T HM contained slightly higher fat levels, which also effectively matches published data [10]. 

Concerning the lactose content, we observed stable concentrations over time with a trend of higher content in the PT versus T HM. Our results are in line with most of the previous reported data, although some studies described an increase in lactose concentration during the first month of lactation [10,35,39]. As lactose constitutes the major energy source among total carbohydrates, it is an acceptable proxy for digestible carbohydrates [35].

Finally, the energy density was not directly measured in our study, but it was calculated and corrected for adjusted protein content for each milk sample, using the formula published by Polberg and Lönnerdal [30]. Overall, values of our cohort tended to be generally slightly lower than those reported in the literature [10,35,39]. The single morning HM sampling procedure could have contributed to this observation, as fat content has been reported to be higher in the evening [5]. 

Besides these temporal factors of variations, we also investigated the influence of other factors, such as gender and mode of delivery. We noticed that HM for male infants was more concentrated in fat and energy as compared with female infants. This was observed both in the T and PT group, but it was more marked in the PT group. A multivariate analysis confirmed that the infant’s male gender was significantly and independently associated with higher concentrations of fat and energy in HM. So far, this phenomenon of “sex bias” milk synthesis, possibly already partly conditioned in utero, has mainly been studied and discussed in other mammals, such as cows, horses, monkeys, and hamsters [40,41]. In humans, by contrast, only a small number of studies have reported a possible gender specificity of the HM composition. In 2010, Powe et al. observed an increase in energy value of 25% for male infants of well-fed American women [42]. In a study conducted in Singapore, Thakkar et al. found similar differences in term newborns (energy + 24% and fat + 39% for boys), with a lipid profile that also varied by gender [43]. More recently, in a term delivering population of mothers living in Seoul, Hahn also observed an increased energy density in HM for male infants, related to a higher carbohydrates content. By contrast, Quinn et al. did not observe any gender difference in the milk of 103 Filipino women [7,44]. Interaction with environmental and/or nutritional conditions is possible, as suggested by Fujita et al. [45]. According to an evolutionary perspective, these authors hypothesize that economically disadvantaged mothers produce richer milk for girls, while well-nourished women produce richer milk for boys [45]. According to BMI indicators, our population was rather well nourished [29]. The reasons for these observed differences remain unclear. One can hypothesize that, due to their different growth trajectories and hormonal environment, male and female infants may have different energy requirements. The implications of such differences, if confirmed, could be important. On the other hand, the effect of delivery mode reported by Dizdar et al. was not verified in our population [8]. However, due to the relatively limited number of subjects and the important variability of data, these results should be interpreted cautiously. 

This work has some limitations to consider: (i) as mentioned above, the method of sampling (first milk of the morning rather than pooled over 24 h) could have increased the measured variations, especially for lipids; (ii) variations in milk volumes, that may have also contributed to the important variability observed, were not recorded and thus could not be taken into account; (iii) the method of measuring macronutrients (MIRIS ©) is a method whose reliability is open to criticism [46]. We attempted to enhance its accuracy through validation methods and protein correction strategy [31]; (iv) finally, the small number of patients in each group limited the power of the study, and allowed only a limited number of associated factors to be analyzed. However, the influence of other factors, such as intrauterine growth restriction, twinning, antenatal steroids, smoking, and maternal comorbidities, for example, also deserves to be further investigated.

Despite this, our results emphasize that, although pragmatic, a unique definition of a “standard” nutritional composition of HM, as traditionally applied, can be insufficient and inaccurate for the nutritionally vulnerable infants, such as preterm infants [47]. These infants may require more individualized nutritional approaches, such as “targeted” fortification based on regular measurements of HM composition, or “adjusted” fortification according to serum urea values [11,30,48]. However, so far, targeted approaches remain challenging and expensive in daily care practices, without enthusiastic durable results on growth or development. Thus, future research should aim to develop optimized, efficient and feasible fortification strategies. Recent progresses in bioengineering should help in these issues [49]. Importantly, studies should also investigate other maternal and infant factors interacting with HM composition, including offspring gender. 

Meanwhile, it would be good to consider changing the way nutritional intakes are calculated for clinical or research purposes. Currently, when direct measurements of HM composition are not available, a unique HM composition is generally assumed. However, it would be preferable to use a model that adjusts the assumed composition according to the T/PT status and/or postnatal age as recently proposed by an expert group [34].

## 5. Conclusions

Macronutrient composition and energy density in human milk change over time, with important inter- and intra- individual variations. The original design of this longitudinal study allowed us to compare term and preterm mother milk composition both at similar postnatal stages, corresponding to similar adaptive stages after birth, and at similar gestational ages, corresponding to similar developmental stages after conception. This original approach revealed that differences between the PT and T HM were more preponderant at equivalent gestational ages, especially around term, than at equivalent postpartum lactation stages. Moreover, our results suggest a possible gender adaptation of HM, with a more caloric milk for male infants. This is also a rather novel and compelling issue that deserves additional research.

Accordingly, there is definitely not one, but a multitude of human milk compositions and breastfeeding appears to be a powerful preventive and personalized medicine. Whereas HM use is strongly encouraged in preterm as well as in term infants, there is a need to further explore HM composition and variations to assess whether more individualized nutritional approaches may help in optimizing growth and development of more vulnerable infants.

## Supplementary Materials

The following are available online at https://www.mdpi.com/2072-6643/11/7/1525/s1, Figure S1: Study flow chart, Figure S2: Macronutrients by gender, Figure S3: Macronutrients by delivery, Table S1: Maternal and infant characteristics, Table S2: Macronutrient data: postnatal, postmenstrual, gender, delivery.

## References

[1] Andreas N.J., Kampmann B., Mehring Le-Doare K. (2015). Human breast milk: A review on its composition and bioactivity. Early Hum. Dev..

[2] Jakaitis B.M., Denning P.W. (2014). Human breast milk and the gastrointestinal innate immune system. Clin. Perinatol..

[3] Ballard O., Morrow A.L. (2013). Human milk composition: Nutrients and bioactive factors. Pediatr. Clin. N. Am..

[4] Picciano M.F. (2001). Nutrient composition of human milk. Pediatr. Clin. N. Am..

[5] Moran-Lev H., Mimouni F.B., Ovental A., Mangel L., Mandel D., Lubetzky R. (2015). Circadian Macronutrients Variations over the First 7 Weeks of Human Milk Feeding of Preterm Infants. Breastfeed. Med..

[6] Çetinkaya A.K., Dizdar E.A., Yarcı E., Sari F.N., Oguz S.S., Uras N., Canpolat F.E. (2017). Does Circadian Variation of Mothers Affect Macronutrients of Breast Milk?. Am. J. Perinatol..

[7] Quinn E.A. (2013). No evidence for sex biases in milk macronutrients, energy, or breastfeeding frequency in a sample of Filipino mothers. Am. J. Phys. Anthropol..

[8] Dizdar E.A., Sari F.N., Degirmencioglu H., Canpolat F.E., Oguz S.S., Uras N., Dilmen U. (2014). Effect of mode of delivery on macronutrient content of breast milk. J. Matern. Fetal Neonatal Med..

[9] Affolter M., Garcia-Rodenas C.L., Vinyes-Pares G., Jenni R., Roggero I., Avanti-Nigro O., de Castro C.A., Zhao A., Zhang Y., Wang P. (2016). Temporal Changes of Protein Composition in Breast Milk of Chinese Urban Mothers and Impact of Caesarean Section Delivery. Nutrients.

[10] Gidrewicz D.A., Fenton T.R. (2014). A systematic review and meta-analysis of the nutrient content of preterm and term breast milk. BMC Pediatr..

[11] Rochow N., Landau-Crangle E., Fusch C. (2015). Challenges in breast milk fortification for preterm infants. Curr. Opin. Clin. Nutr. Metab. Care.

[12] Gibertoni D., Corvaglia L., Vandini S., Rucci P., Savini S., Alessandroni R., Sansavini A., Fantini M.P., Faldella G. (2015). Positive effect of human milk feeding during NICU hospitalization on 24 month neurodevelopment of very low birth weight infants: An Italian cohort study. PLoS ONE.

[13] Section on Breastfeeding (2012). Breastfeeding and the use of human milk. Pediatrics.

[14] WHO (2007). Evidence on the Long-Term Effects of Breastfeeding. http://www.who.int/maternal_child_adolescent/documents/9241595230/en/.

[15] Edmond K., Bahl R., WHO Optimal Feeding of Low-Birth-Weight Infants. http://www.who.int/maternal_child_adolescent/documents/9241595094/en/.

[16] Moro G.E., Arslanoglu S., Bertino E., Corvaglia L., Montirosso R., Picaud J.C., Polberger S., Schanler R.J., Steel C., van Goudoever J. (2015). XII. Human Milk in Feeding Premature Infants: Consensus Statement. J. Pediatr. Gastroenterol. Nutr..

[17] Miller J., Tonkin E., Damarell R.A., McPhee A.J., Suganuma M., Suganuma H., Middleton P.F., Makrides M., Collins C.T. (2018). A Systematic Review and Meta-Analysis of Human Milk Feeding and Morbidity in Very Low Birth Weight Infants. Nutrients.

[18] Boquien C.Y. (2018). Human Milk: An Ideal Food for Nutrition of Preterm Newborn. Front. Pediatr..

[19] Underwood M.A. (2013). Human milk for the premature infant. Pediatr. Clin. N. Am..

[20] Menon G., Williams T.C. (2013). Human milk for preterm infants: Why, what, when and how?. Arch. Dis. Child. Fetal Neonatal Ed..

[21] Vohr B.R., Poindexter B.B., Dusick A.M., McKinley L.T., Higgins R.D., Langer J.C., Poole W.K., National Institute of Child Health and Human Development National Research Network (2007). Persistent beneficial effects of breast milk ingested in the neonatal intensive care unit on outcomes of extremely low birth weight infants at 30 months of age. Pediatrics.

[22] Belfort M.B., Anderson P.J., Nowak V.A., Lee K.J., Molesworth C., Thompson D.K., Doyle L.Y., Inder T.F. (2016). Breast Milk Feeding, Brain Development, and Neurocognitive Outcomes: A 7-Year Longitudinal Study in Infants Born at Less Than 30 Weeks’ Gestation. J. Pediatr..

[23] Belfort M.B., Ehrenkranz R.A. (2017). Neurodevelopmental outcomes and nutritional strategies in very low birth weight infants. Semin. Fetal Neonatal Med..

[24] Blesa M., Sullivan G., Anblagan D., Telford E.J., Quigley A.J., Sparrow S.A., Serag A., Semple S.I., Bastin M.E., Boardman J.P. (2018). Early breast milk exposure modifies brain connectivity in preterm infants. NeuroImage.

[25] Henderson G., Anthony M.Y., McGuire W. (2007). Formula milk versus maternal breast milk for feeding preterm or low birth weight infants. Cochrane Database Syst. Rev..

[26] Kuschel C.A., Harding J.E. (2004). Multicomponent fortified human milk for promoting growth in preterm infants. Cochrane Database Syst. Rev..

[27] Tudehope D.I. (2013). Human milk and the nutritional needs of preterm infants. J. Pediatr..

[28] Horbar J.D., Ehrenkranz R.A., Badger G.J., Edwards E.M., Morrow K.A., Soll R.F., Buzas J.S., Bertino E., Gagliardi L., Bellù R. (2015). Weight Growth Velocity and Postnatal Growth Failure in Infants 501 to 1500 Grams: 2000–2013. Pediatrics.

[29] Garcia-Rodenas C.L., De Castro C.A., Jenni R., Thakkar S.K., Beauport L., Tolsa J.F., Fischer-Fumeaux C.J., Affolter M. (2018). Temporal changes of major protein concentrations in preterm and term human milk. A prospective cohort study. Clin. Nutr. Edinb. Scotl..

[30] Polberger S., Lönnerdal B. (1993). Simple and rapid macronutrient analysis of human milk for individualized fortification: Basis for improved nutritional management of very-low-birth-weight infants?. J. Pediatr. Gastroenterol. Nutr..

[31] Giuffrida F., Austin S., Cuany D., Sanchez-Bridge B., Longet K., Bertschy E., Sauser J., Thakkar S.K., Lee L.Y., Affolter M. (2018). Comparison of macronutrient content in human milk measured by mid-infrared human milk analyzer and reference methods. J. Perinatol..

[32] Cooper A.R., Barnett D., Gentles E., Cairns L., Simpson J.H. (2013). Macronutrient content of donor human breast milk. Arch. Dis. Child. Fetal Neonatal. Ed..

[33] García-Lara N.R., Escuder-Vieco D., García-Algar O., De la Cruz J., Lora D., Pallás-Alonso C. (2012). Effect of freezing time on macronutrients and energy content of breastmilk. Breastfeed. Med..

[34] Cormack B.E., Embleton N.D., van Goudoever J.B., Hay W.W., Bloomfield F.H. (2016). Comparing apples with apples: It is time for standardized reporting of neonatal nutrition and growth studies. Pediatr. Res..

[35] Boyce C., Watson M., Lazidis G., Reeve S., Dods K., Simmer K., McLeod G. (2016). Preterm human milk composition: A systematic literature review. Br. J. Nutr..

[36] Hsu Y.C., Chen C.H., Lin M.C., Tsai C.R., Liang J.T., Wang T.M. (2014). Changes in preterm breast milk nutrient content in the first month. Pediatr. Neonatol..

[37] Bauer J., Gerss J. (2011). Longitudinal analysis of macronutrients and minerals in human milk produced by mothers of preterm infants. Clin. Nutr. Edinb. Scotl..

[38] Maly J., Burianova I., Vitkova V., Ticha E., Navratilova M., Cermakova E., Premature Milk Study Group (2019). Preterm human milk macronutrient concentration is independent of gestational age at birth. Arch. Dis. Child. Fetal Neonatal Ed..

[39] Tsang R., Uauy R., Koletzko B., Zlotkin S. (2005). Nutrition of the preterm infant. Early Hum. Dev..

[40] Hinde K. (2009). Richer milk for sons but more milk for daughters: Sex-biased investment during lactation varies with maternal life history in rhesus macaques. Am. J. Hum. Biol..

[41] Hinde K., Carpenter A.J., Clay J.S., Bradford B.J. (2014). Holsteins favor heifers, not bulls: Biased milk production programmed during pregnancy as a function of fetal sex. PLoS ONE.

[42] Powe C.E., Knott C.D., Conklin-Brittain N. (2010). Infant sex predicts breast milk energy content. Am. J. Hum. Biol..

[43] Thakkar S.K., Giuffrida F., Cristina C.H., De Castro C.A., Mukherjee R., Tran L.A., Steenhout P., Lee L.Y., Destaillats F. (2013). Dynamics of human milk nutrient composition of women from Singapore with a special focus on lipids. Am. J. Hum. Biol..

[44] Hahn W.H., Song J.H., Song S., Kang N.M. (2017). Do gender and birth height of infant affect calorie of human milk? An association study between human milk macronutrient and various birth factors. J. Matern. Fetal Neonatal Med..

[45] Fujita M., Roth E., Lo Y.J., Hurst C., Vollner J., Kendell A. (2012). In poor families, mothers’ milk is richer for daughters than sons: A test of Trivers-Willard hypothesis in agropastoral settlements in Northern Kenya. Am. J. Phys. Anthropol..

[46] Fusch G., Rochow N., Choi A., Fusch S., Poeschl S., Ubah A.O., Lee S.Y., Raja P., Fusch C. (2015). Rapid measurement of macronutrients in breast milk: How reliable are infrared milk analyzers?. Clin. Nutr. Edinb. Scotl..

[47] Kleinman R.E., American Academy of Pediatrics. Committee on Nutrition (2004). Nutritional Needs of Preterm Infants. Pediatric Nutrition Handbook.

[48] Arslanoglu S. (2015). IV. Individualized Fortification of Human Milk: Adjustable Fortification. J. Pediatr. Gastroenterol. Nutr..

[49] Van Goudoever J. (2015). VI. Bioengineering Human Milk: Why?. J. Pediatr. Gastroenterol. Nutr..

