# Supplementary material for: Longitudinal Analysis of Macronutrient Composition in Preterm and Term Human Milk: A Prospective Cohort Study
Source: Nutrients. 2019 Jul 4;11(7):1525. doi: 10.3390/nu11071525 (PMC6683284; doi:10.3390/nu11071525)
Supplement: Supplementary file 1 [file nutrients-11-01525-s001.zip › Table S1 - Maternal and infant characteristics.pdf]

Table S1. Maternal and infant characteristics.

Study population	Preterm	Term	*p*-value
**Mother**	***n* = 25**	***n* = 28**	
Age (years), mean ± SD	32.4 ± 5.6	31.2 ± 4.2	0.3173
Height (cm), mean ± SD	165.2 ± 7.1	166.8 ± 6.6	0.3601
Weight before pregnancy (kg), mean ± SD	62.1 ± 9.5	64.3 ± 12.0	0.4479
Weight at birth (kg), mean ± SD	70.3 ± 10.6	74.5 ± 11.3	0.1426
BMI before pregnancy (kg/m^2), mean ± SD	22.8 ± 3.4	23.2 ± 4.9	0.6990
BMI at birth (kg/m^2), mean ± SD	25.8 ± 3.7	26.9 ± 4.7	0.3141
Caesarean delivery, *N* (%)	63.0	23.5	0.0019
**Infant**	***n* = 33**	***n* = 34**	
Gestational age at birth (weeks) , mean ± SD	30.8 ± 1.4	39.5 ± 1.0	<0.0001
Males, *N* (%)	54.5	52.9	0.8952
Twins, *N* (%)	36.4	0.0	0.0001
Birth weight (g), mean ± SD	1421.4 ± 372.8	3277.6 ± 353.6	<0.0001
Birth height (cm), mean ± SD	40.4 ± 3.2	49.4 ± 1.7	<0.0001
Birth head circumference (cm), mean ± SD	27.8 ± 2.1	34.4 ± 1.5	<0.0001

(reprinted from Garcia-Rodenas et al. 2018, with permission from Elsevier)
