# Supplementary figures and images for: Longitudinal Analysis of Macronutrient Composition in Preterm and Term Human Milk: A Prospective Cohort Study
Source: Nutrients. 2019 Jul 4;11(7):1525. doi: 10.3390/nu11071525 (PMC6683284; doi:10.3390/nu11071525)

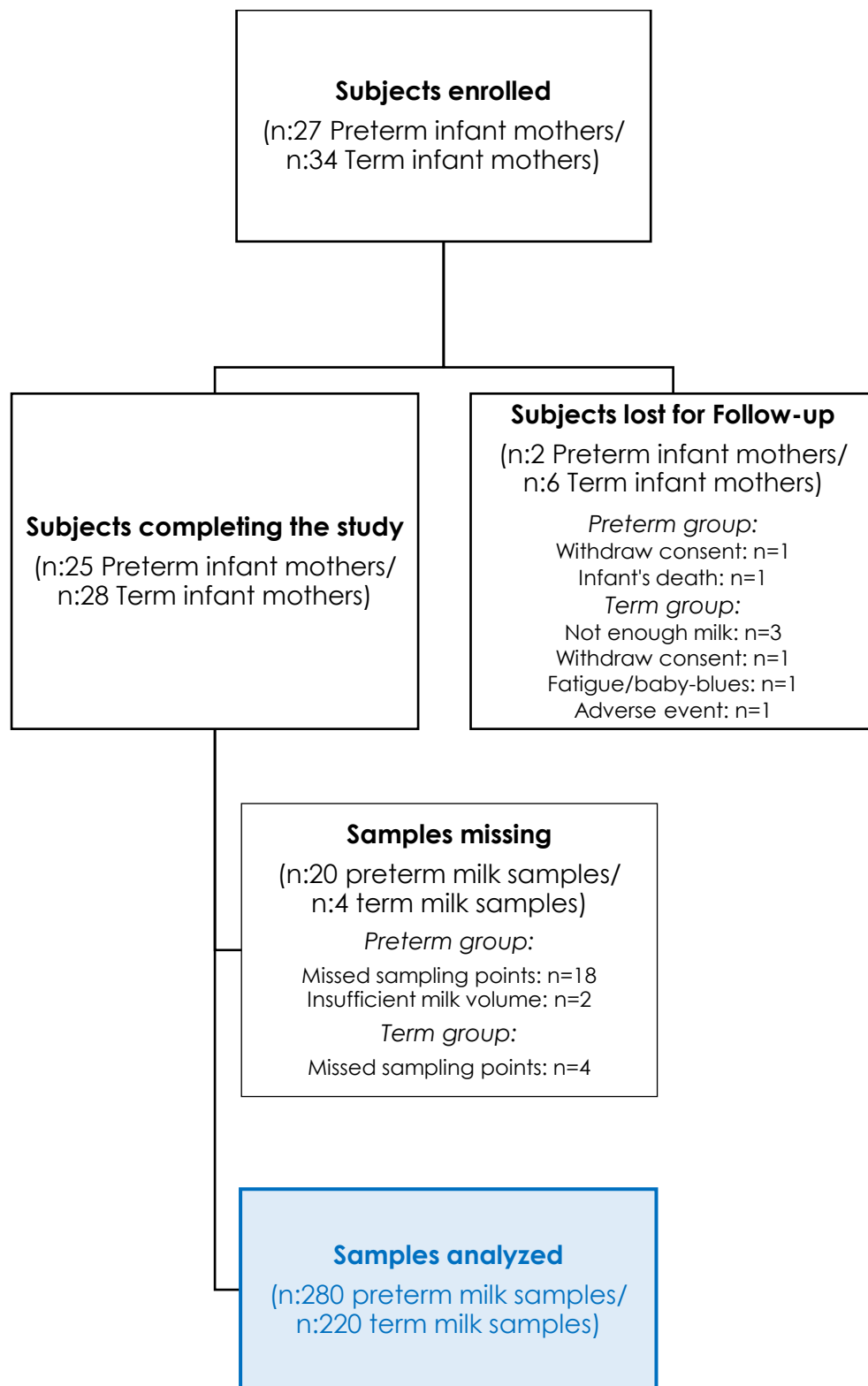

**Figure S1.** Study flow chart

(reprinted from Garcia-Rodenas et al. 2018, with permission from Elsevier)

Supplement: Supplementary file 1 [file nutrients-11-01525-s001.zip › Figure S1 - Study Flow Chart.pdf]

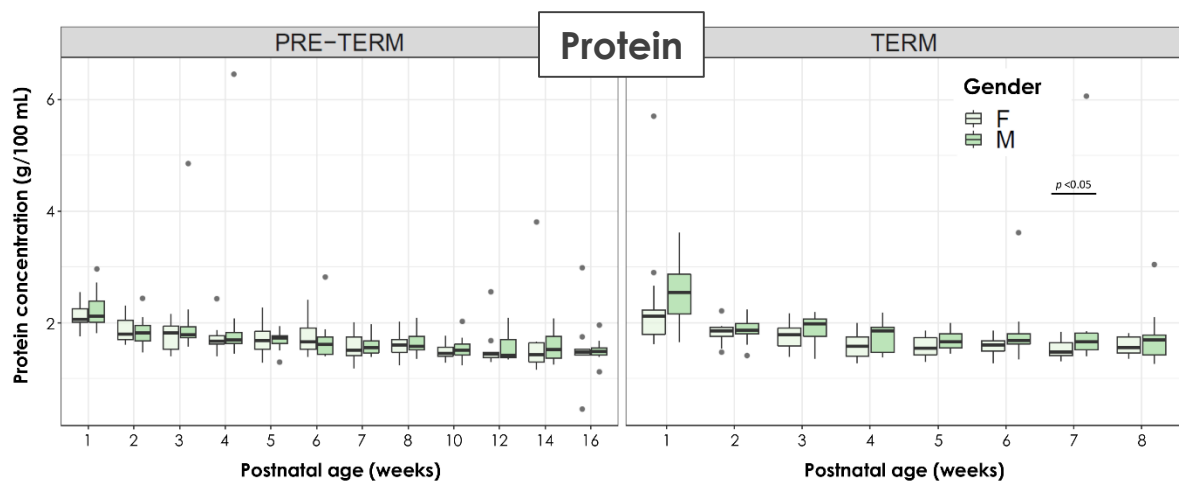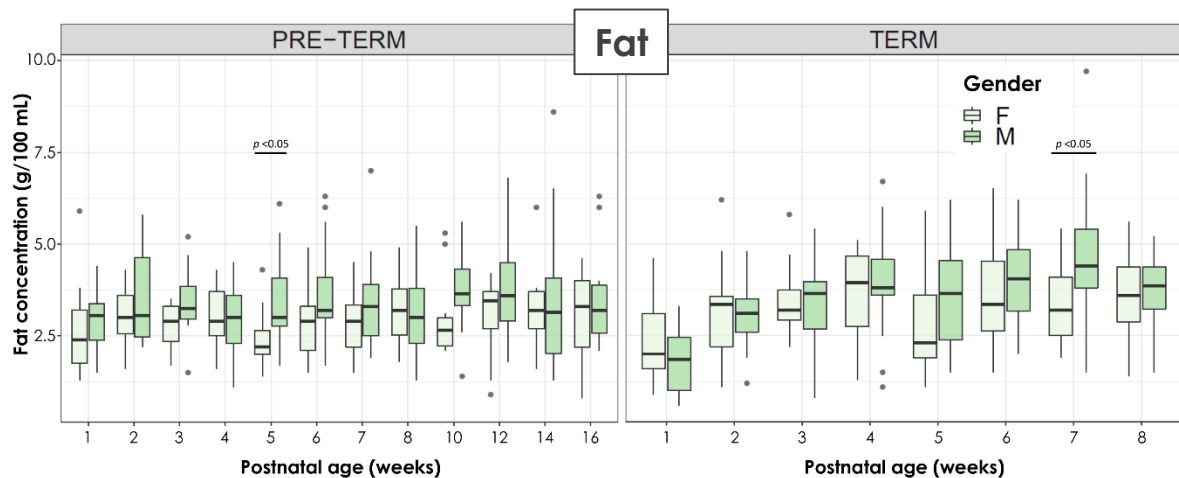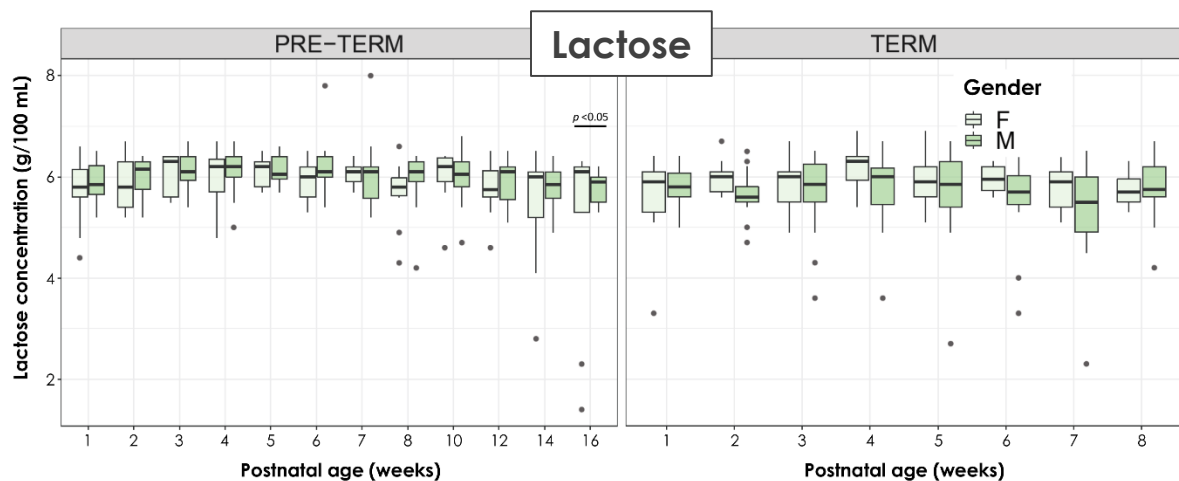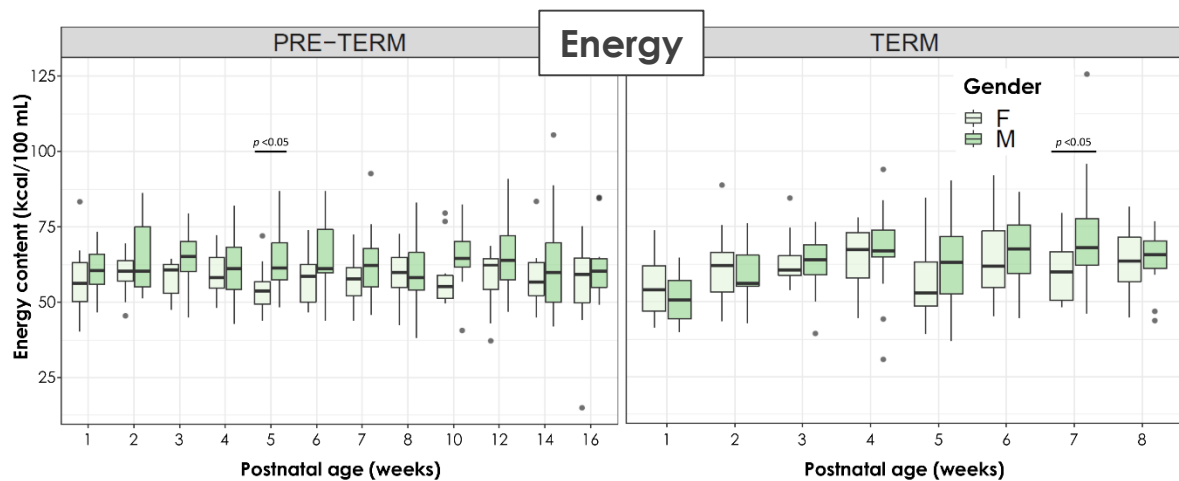

Supplement: Supplementary file 1 [file nutrients-11-01525-s001.zip › Figure S2 - Macronutrients_by_gender.pdf]

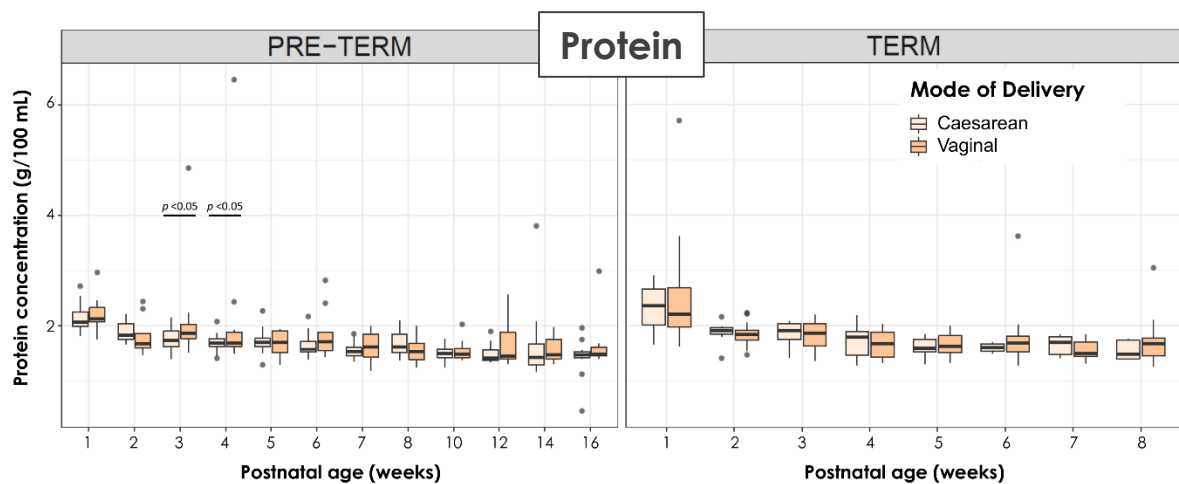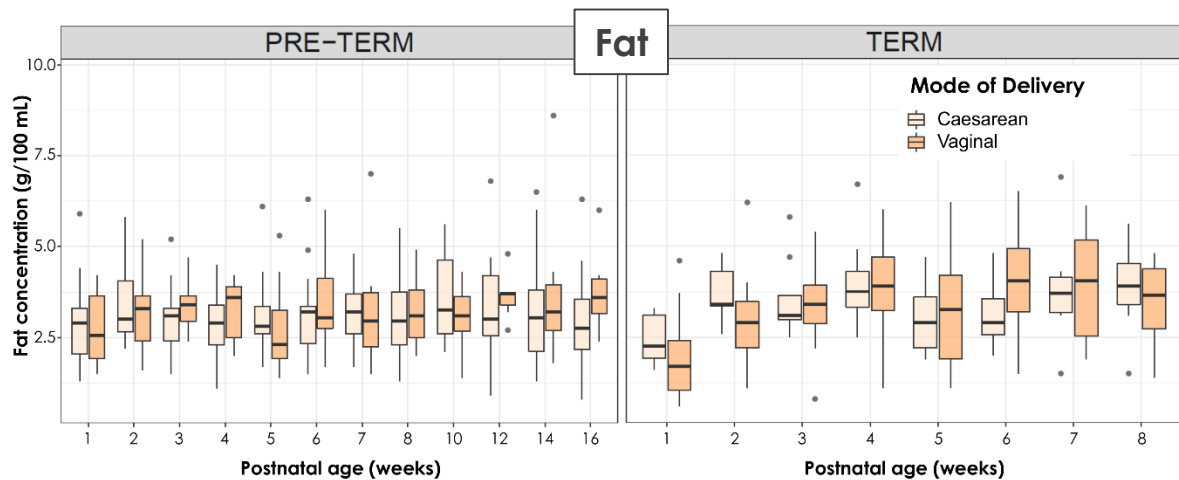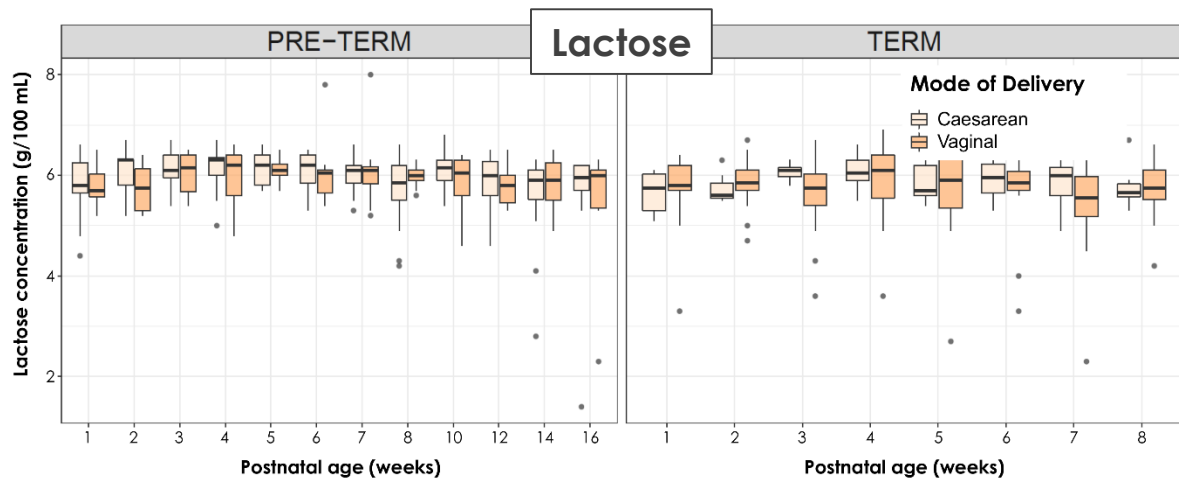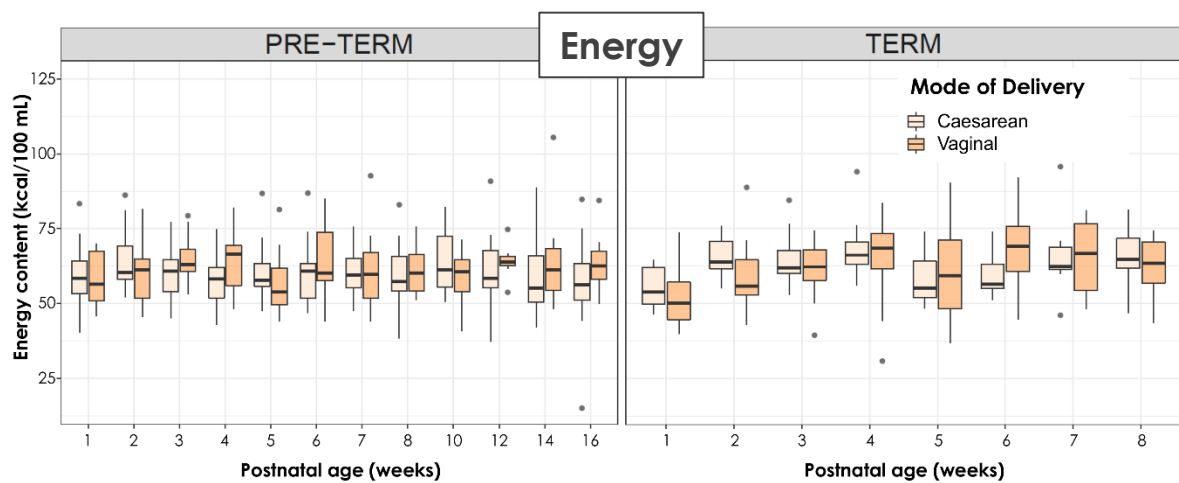

Supplement: Supplementary file 1 [file nutrients-11-01525-s001.zip › Figure S3 - Macronutrients_by_delivery.pdf]
